# Embodiment and sense-making in autism

**DOI:** 10.3389/fnint.2013.00015

**Published:** 2013-03-26

**Authors:** Hanne De Jaegher

**Affiliations:** ^1^Department of Logic and Philosophy of Science, IAS-Research Centre for Life, Mind, and Society, University of the Basque CountrySan Sebastián, Spain; ^2^Department of Informatics, Centre for Computational Neuroscience and Robotics, Centre for Research in Cognitive Science, School of Life Sciences, University of SussexBrighton, UK

**Keywords:** autism, enaction, sense-making, participatory sense-making, embodiment, social interaction, coordination dynamics

## Abstract

In this article, I sketch an enactive account of autism. For the enactive approach to cognition, embodiment, experience, and social interaction are fundamental to understanding mind and subjectivity. Enaction defines cognition as sense-making: the way cognitive agents meaningfully connect with their world, based on their needs and goals as self-organizing, self-maintaining, embodied agents. In the social realm, the interactive coordination of embodied sense-making activities with others lets us participate in each other's sense-making (social understanding = participatory sense-making). The enactive approach provides new concepts to overcome the problems of traditional functionalist accounts of autism, which can only give a piecemeal and disintegrated view because they consider cognition, communication, and perception separately, do not take embodied into account, and are methodologically individualistic. Applying the concepts of enaction to autism, I show:

1. How embodiment and sense-making connect, i.e., how autistic particularities of moving, perceiving, and emoting relate to how people with autism make sense of their world. For instance, restricted interests or preference for detail will have certain sensorimotor correlates, as well as specific meaning for autistic people.
2. That reduced flexibility in interactional coordination correlates with difficulties in participatory sense-making. At the same time, seemingly irrelevant “autistic behaviors” can be quite attuned to the interactive context. I illustrate this complexity in the case of echolalia.

An enactive account of autism starts from the embodiment, experience, and social interactions of autistic people. Enaction brings together the sensorimotor, cognitive, social, experiential, and affective aspects of autism in a coherent framework based on a complex non-linear multi-causality. This foundation allows to build new bridges between autistic people and their often non-autistic context, and to improve quality of life prospects.

## Introduction

Autism is primarily seen as a combination of social, communicative, and cognitive deficits. However, there is growing awareness that autism is also characterized by different ways of perceiving and moving, as well as particular emotional-affective aspects. Evidence ranges from hypo- and hyper-sensitivities, over difficulties with the timing, coordination, and integration of movement and perception, painfulness of certain stimuli, muscle tone differences, rigid posture, movement, attention, and saliency problems, to differences in bodily coordination during social interactions.

If the social, communicative, and cognitive deficits were difficult to pull together under one explanation, now, as the many and divergent aspects of what we may call autistic embodiment are gaining interest, an integrative explanation seems still further off.

In this paper, I explain why three of the main autism theories [theory of mind (ToM), weak central coherence (WCC), and executive function (EF)] are inherently piecemeal, and why this is a problem.

Then, I sketch a proposal to bring the many aspects of autism together, by doing justice to the experience of autism. The proposal is based on the enactive approach to cognition, which uses the notion of *sense-making* to define cognition as the meaningful way in which an agent connects with her world. It brings a dimension of personal significance right to the core of cognition. Sense-making is based in the inherent needs and goals that come with being a bodily, self-organizing, self-maintaining, precarious being with a singular perspective on the world. Sense-making plays out and happens through the embodiment and situatedness of the cognitive agent: her ways of moving and perceiving, her affect and emotions, and the context in which she finds herself, all determine the significance she gives to the world, and this significance in turn influences how she moves, perceives, emotes, and is situated.

The social side of this—important in cognition in general, and also for understanding autism—is captured by the notion of *participatory sense-making*, which describes how individual sense-making is affected by inter-individual coordination. If sense-making is a thoroughly embodied activity, and we can coordinate our movements, perceptions, and emotions in interactions with each other, then, in social situations, we can literally *participate in each other's sense-making*. This notion brings the dynamics of interactive encounters into the foreground and provides novel elements for the study of autism, such as the idea of the rhythmic capacity (discussed below). The notion connects ways of measuring coordination (using dynamical systems tools) with the investigation of the 1st and 2nd person experience of autism.

These are the central items that I apply to autism research in order to uncover the relation between what I call “autistic embodiment” and “autistic psychology.” On the basis of empirical research, I show that autism is characterized by a different embodiment, and propose hypotheses based on the dimensions of significance that are inherent in sense-making. I suggest that their great attention to detail, preference for repetition and sameness, and restricted interests may be inherently meaningful for people with autism, and not just, as they have often been conceived, inappropriate behaviors to be treated away. In the social and communication realm, I suggest that social interaction difficulties are not to be considered exclusively as individually based, but that the patterns in the interaction processes that autistic people engage in play an important role in them. Evidence shows that people on the spectrum have difficulties with temporal coordination in social interactions, but also unexpected capacities in this area. I propose that people with autism are less flexible in dealing with the wide range of interactional styles that characterize social life, but that how they can deal with this depends not just on individual capacities, but also on the interactions they engage in. Different measurable aspects of the dynamics of interactions involving people with autism illustrate this. Finally, I discuss some implications for diagnosis, remediation, integration, and quality of life.

## Current understanding of autism

### Three theories

Autism is most often seen as a combination of social, communicative, and cognitive deficits. The three explanatory theories addressing these aspects are ToM, WCC, and EF (Baron-Cohen, 2003; Frith, 2003).

ToM theory aims to explain non-autistic social cognition in functional/computational terms. Underlying it is the assumption that what other people think and feel is internal and hidden from us, and the only clue we have to go on is their perceptible behavior. From this, we supposedly infer their intentions, using dedicated neural and/or cognitive mechanisms. People with autism are thought to have more trouble than usual doing this, and to find it difficult to “read other people's minds,” or to imagine what they are thinking or feeling. The suggestion is that autistic people lack or have a broken “ToM”—the purported neural or cognitive device that computes others' underlying intentions from their perceived behaviors—or to have difficulties with “mindreading” or “mentalizing” (Baron-Cohen et al., 1985, 1986; Baron-Cohen, 1995; Goldman, 2012). This proposal underlies much of the traditional understanding of autism, and has been very fruitful in terms of research output. It has been around since the 1970s, and many studies today, not just of autism but of social cognition generally, are still built on its basis (see e.g., Sterck and Begeer, 2010), although more recent findings also suggest that people with autism tend to be better at mindreading than thought before (see e.g., Begeer et al., 2010; Lombardo et al., 2010; Roeyers and Demurie, 2010; see also Happé, 1994).

WCC theory (Frith, 1989; Shah and Frith, 1993; Frith and Happé, 1994; Happé, 1994) suggests that people with autism focus on piecemeal information and have difficulty integrating what they perceive as well as perceiving things in context. This difficulty is manifested at different levels, from perceiving whole objects to grasping the gist of a story. For example, research shows that it is difficult to read homographs in context (Frith and Snowling, 1983; Happé, 1997; López and Leekam, 2003). Francesca Happé, Uta Frith and others also call WCC a cognitive *style* (Happé, 1999; Frith, 2003). Neurotypicals^1^ tend to prefer processing the overall meaning of a scene, while autistics focus on details. WCC research has generated interest in remarkable aspects of autistic perception, and has given attention to what can be seen not just as deficits, but as cognitive capacities and advantages (Happé, 1999; Frith, 2003; Happé and Frith, 2009; Mottron, 2011). Some of the more striking such capacities are making jigsaw puzzles upside down or without a picture on it (Frith and Hermelin, 1969; Frith, 1989, 2003), or finding hidden figures, e.g., triangles, in a drawing of an object like a house or baby cot (Shah and Frith, 1983).

The EF theory proposes that people with autism lack control over their actions and attention, associated with activity in the frontal lobes. This would explain, for instance, problems with the inhibition of behavior, the strong need for routines and structure, narrow interests, repetitive and stereotypic movements and thought processes, and a need for sameness (Ozonoff et al., 1991; Russell et al., 1991; Russell, 1998). It predicts that people with autism have difficulties with, for instance, the Stroop test, which assesses inhibition, and the Tower of London test, which evaluates planning capacities (Robinson et al., 2009).

### Problems with the theories

These theories are not without problems. For instance, Boucher (2012) argues that ToM is too focused on high-level capacities, while it is not clear what could be underlying them. Also, while some people with autism do pass ToM tests, some with other disabilities (and not autism) do not pass high-level ToM tests, leading to the question of whether ToM deficits reliably pick out autism in particular (see e.g., Happé, 1994; Boucher, 2012). If language abilities and higher order reasoning are closely intertwined with ToM (Sigman et al., 1995; for a discussion, see Malle, 2002), maybe autism is rather a problem with language and reasoning? Or could it even be that people on the spectrum, good as they seem to be at literal reasoning, and strict application of structures and rules, are in fact the ones who do use an explicit ToM? As Sigman et al. (1995) found, there may be a connection between high reasoning capacities and good scores on ToM tests in people with autism, because they can “calculate” ToM-like inferences and explanations of behavior. Despite this, such calculations seem to have a limited effect since teaching people with autism about the “rules” of social interaction and perception does not necessarily lead to greater social fluency (Ozonoff and Miller, 1995).

WCC has been criticized for being overly focused on a deficit at the level of contextual, global processing, while there is also research showing a preference for local processing, with global processing sometimes intact (see e.g., Plaisted et al., 1999; Mottron et al., 2000). The theory is also questioned on the basis of how central the drive for central coherence really is, i.e., whether a deficit in integrated information processing spans all levels of cognitive processing (López et al., 2008).

Regarding EF (like for ToM), it is not clear that it is specific to autism and not other disorders, whether all people with autism have executive function deficits, and also precisely how such deficits develop (Hill, 2004a,b).

Perhaps more important than the specific criticisms is the fact that none of the theories suffices on its own to explain autism as a whole. While ToM explains the so-called triad symptoms: social, communicative, and cognitive deficits (Wing and Gould, 1979; American Psychiatric Association, 2000), WCC addresses the non-triad symptoms (narrow attention to detail, islets of ability, and context-insensitivity), and EF deals with the repetitive behaviors (Baron-Cohen, 2003; Frith, 2003). Frith argues that autism is such a complex phenomenon that it needs all these theories (Frith, 2003). She proposes to unify them by searching for the common denominator in the key symptoms of autism, which she suggests is an “absent self” or a lack of top-down control. Frith invokes the age-old idea of the homunculus to explain this. The homunculus—Latin for “little man”—is a kind of controller in the brain, who views what comes in from the sense organs, interprets the situation using these signals, and then sends commands to the muscles and executive organs, so that the human can react appropriately. The idea has a troubled history in philosophy and psychology, and many reject it altogether (Bennett and Hacker, 2003). One of the reasons is that another “little man” inside the first one would be needed to control *his* brain states, and then another one inside the latter one, and so on, *ad infinitum* (see, for instance, Dreyfus, 1992). While recognizing this problem, but also that the idea of a homunculus is, indeed, nevertheless taken for granted in much of neuroscience and psychology, Frith suggests that maybe there is an ultimate homunculus, one behind or inside of which there is not a further one anymore. She proposes that this final homunculus is self-awareness or the ultimate controller, and that this is what people with autism lack (Frith, 2003). How this might be possible is not explained. Major theoretical difficulties aside, evidence supporting such an idea is anything but conclusive. And even then, it is not clear how this lack would explain all the aspects of autism (Frith, 2008).

### Disembodied and isolated

When taking a step back and looking at these theories with some distance, we notice in all of them two important under-considered elements. Firstly, they show little concern for the embodiment and situatedness of the autistic person, and secondly, even in the investigation of social deficits, interactive factors do not play an explanatory role. The theories are disembodied and methodologically individualistic.

The domination of functionalist explanations of autism—at least in the Anglo-Saxon research world—has left other significant aspects of autism all but ignored (or at best informally recognized but never making an impact on research, which amounts to the same). Lately, however, there is increasing interest in the different ways of moving, perceiving, and emoting of autistic people. There is more and more research on autistic perception, hypo- and hyper-sensitivities, movement, and emotional specificities (Gepner et al., 1995, 2001; Baranek, 2002; Gepner and Mestre, 2002a; Rogers and Ozonoff, 2005; Fournier et al., 2010; Whyatt and Craig, 2012; Donnellan et al., 2013; Smith and Sharp, in press).

The embodied turn in cognitive science urges us to take the role of the body in subjectivity and cognition seriously (see Brooks, 1991; Varela et al., 1991; Dreyfus, 1992; Lakoff and Johnson, 1999; O'Regan and Noë, 2001; Gallagher, 2005; Thompson, 2007; Gallagher and Zahavi, 2008, etc.). Embodied approaches agree that the body plays a crucial role in cognition and emotion. They vary, however, as to the role for and notions of the body they propose. For extended functionalism, the body primarily simplifies cognitive information processing, “offloading” it from brain to muscles (Clark and Chalmers, 1998; Wheeler, 2010). For the sensorimotor approach, perceptual experience and cognition are grounded in the mastering of regularities in sensorimotor activity that depends on bodily structures and habits (O'Regan and Noë, 2001). For enaction, the body may play the above roles but it is in addition an organic precarious self-sustaining system with needs, and this is why embodied creatures care about their world in the first place, they have their own perspective of significance which is rooted in the body (Varela et al., 1991; Thompson, 2007; Di Paolo et al., 2010). It is this approach that forms the basis for the view on autism that I take here.

Furthermore, the trio of theories, while they are centrally concerned with autism's most striking difficulty—its social and communicative aspects—do not do justice to the possible roles played by social interaction processes (Gallagher, 2001, 2004a; McGeer, 2001). The study of social interaction processes has recently become prominent in social neuroscience, psychology, and developmental psychology (Reddy et al., 1997; Reddy and Morris, 2004; Sebanz et al., 2006; De Jaegher et al., 2010; Dumas et al., 2010; Schilbach, 2010; Di Paolo and De Jaegher, 2012; Pfeiffer et al., 2013; Schilbach et al., in press). Proponents of ToM will say that social interaction is *of course* central to their theory (Michael, 2011). But this is not so obvious. ToM is certainly concerned with social interaction, but only as an input to or an end-product of individual, brain-based, high-level cognitive processes, not as complex processes in their own right or in any of their relevant dynamic features. None of the mainstream theories provides an account of the role that interaction processes *as such* play in how autism manifests, develops, and affects the people on the spectrum as well as those around them.

What is an “interaction as such”? Let me illustrate it with some examples from everyday life. There is a way in which the interactions we engage in can take on a life of their own. This happens, for example, when we feel “in sync” with someone, or when two people speaking on the phone cannot seem to hang up, even if they both feel this is the end of the conversation (Torrance and Froese, 2011), or in cases of interactions that time and again manifest a certain atmosphere, e.g., of animosity, or of flirting—even if each participant clearly wants and even tries to avoid this dynamic (see also Granic, 2000). In these examples, the interaction process, in its extra-individual dimension, influences, modifies, and in part creates the intentions of those engaged in it (De Jaegher and Di Paolo, 2007; De Jaegher, 2009; Fuchs and De Jaegher, 2009; Gallagher, 2009; De Jaegher et al., 2010). Although this plays a great role in everyday life, and also in autism, none of it is accounted for or even considered by ToM, WCC, EF, or any combination of them.

I claim that an integrated theory of autism cannot ignore embodiment and social interaction processes. They are key elements of the enactive account I propose here.

### Limits of piecemeal functionalism

There is another common ill that the three theories suffer. Given their cognitivist and functionalist background, it is no surprise that the accounts consider perception, action, and cognition as relatively separate domains that can be investigated practically in isolation (Frith, 2003; Happé et al., 2006). The overall approach is piecemeal, and the hope is that the insights and explanations will eventually be put together. *How* is another matter. In a way, this is a kind of “weak coherence” view of mind. Or, in the words of Baron-Cohen (though he does not apply this term to autism *theories*), it is a *systemizing* way of thinking, which he associates with male thinking and with autism (Baron-Cohen, 2002), and which is also associated with standard reductionist views of science (see e.g., Polanyi, 1958). Piecemeal approaches can generate partial knowledge, but they have a number of problems at the time of putting the pieces together, especially when the various elements bear intricate relations to each other, as is the case in autism.

First we can ask, what precisely is the link between the different aspects of the “autistic mind”? In general, the aim is for a unified account based on a single causal mechanism or underlying deficit (Volkmar et al., 2004, 2005; though see also Happé et al., 2006, who argue against a single underlying deficit or theory). Functionalism's answers to the question of integration are limited to a linear strategy, in which either everything is reduced to a common root, often a neural function (e.g., Frith's ultimate homunculus), or to a common higher cognitive capacity (e.g., Frith's metaphor of the absent self). But seeking an integrative view of autism does not necessarily imply adopting a mono-causal approach. It can also mean adopting a framework where as many factors as possible cohere, even in the presence of multiple causal elements that relate non-linearly. The analytic, systemizing approach in much of cognitive science and autism research has delivered worthwhile insights, but there is something that remains unclear, something that can only be grasped when we look at all the issues through a synthetic lens too. This something, I suggest, is central to what makes autistic people, and others, relate in meaningful ways with the world. We come back to it below.

We can also ask the question of how the elements of autism are related in specifically developmental terms. The deficits proposed by ToM, WCC, and EF are relatively high-level, and several researchers have pointed out that something is likely to go wrong earlier in development, in so-called precursors to, for instance, a full-blown ToM mechanism (Hobson, 1991, 1993; Klin et al., 1992; Hendriks-Jansen, 1997; Gallagher, 2001, 2004a,b; McGeer, 2001; Hutto, 2003; Zahavi and Parnas, 2003). Often, within these theories, development is thought as the straight temporal sequence between a set of precursors and their concomitant trait. But, as dynamical systems researchers argue, a genuinely developmental approach is one that accounts for change *overtime*, i.e., one that “sees capacities and deficits as not just following each other, but following *from* each other” (Hendriks-Jansen, 1997, p. 383, emphasis in original; see also Fogel, 1993; Thelen and Smith, 1994; Lewis and Granic, 2000; Shanker and King, 2002; Shanker, 2004). On Frith's account of autism, all the problems are tethered to a common anchor, the ultimate self-awareness, which, however, “only gradually emerges in older children and adolescents” (Frith, 2003, p. 209). The fact that the proposed central traits or deficits of autism are relatively high-level makes it difficult to see the developmental trajectory from one symptom to another, let alone how they are meaningfully connected. One keeps wondering: *why* are the symptoms connected in this way? Another way to put this is that, even though research overwhelmingly focuses on *children* with autism^2^, its main explanations are adultist (Sheets-Johnstone, 1999a). That is, they posit adult capacities—or rather, deficits in adult capacities—and then work their way down from there. In this way, it has been hard to imagine that sensory and motor difficulties could be basic to autism, because traditionally it has been hard to imagine the embodied aspects of social reasoning, integrative information processing, planning, or inhibition. The same point can be made about the developmental neglect of social interaction.

If, as I suggest, autism is characterized by differences in embodiment, the question is not just: how do we connect the higher-level psychological functions and traits, but: how do we connect all of this with the differences in perceiving, moving, and emoting? What are the binding factors between autistic embodiment and autistic psychology?

### Toward an enactive account of autism: embodiment, interaction, and experience

Certainly, the criticisms laid out here are all directed at the “dry” theories. This does not preclude scientists, researchers, practitioners, clinicians, teachers, people with autism and their nearest from recognizing, dealing with, and using in their daily practices the elements that I suggest these theories lack. In fact, these people often have a sophisticated intuitive practical understanding of autistic embodiment, behavior, sociality, affect, and experience. However, as long as scientific theories do not describe or explain this know-how, these issues remain poorly understood, poorly connected amongst each other, and difficult to systematically link with practice. Most people who deal with autism in some way or another, whether as a researcher, a practitioner, or personally affected, mean the best, and do their utmost to make life as good as possible with the current knowledge available. But a lot of improvement is still possible and needed, as shown by the fact that even for some of the most integrative and dynamic intervention programs, it is still difficult to bring them to those who need them, or to say why they work (see e.g., Gutstein and Sheely, 2002; Greenspan and Wieder, 2006). Such integrative, holistic programs can use the help of a comprehensive, coherent theory to back them up and provide insight into why certain practices work^3^ and, in turn, the practical know-how of these programs can illuminate and inform theoretical and empirical work.

In sum, I suggest that to understand autism we should avoid piecemeal functionalist pitfalls and their reductionistic demands, while taking stock of the insights that established theories have brought us. An approach that integrates the cognitive, social, communicative, embodied, interactive, experiential, and affective aspects of autism is possible. I propose that this account, based on a coherent and comprehensive view of embodiment, subjectivity, and mind, is *enaction*. In this paper, I can only sketch its potential for understanding autism, and I hope I can at least establish that an integrative understanding of autism—one in which its various elements cohere—requires an account of the embodiment, social interaction processes, and experience of autism.

## Enactive cognitive science

This section provides a necessary and quick introduction to the central concepts of enactive theory. These concepts are applied to autism below, and I introduce them here with a view to this task. I build up the enactive story around two of its main concepts: *sense-making*—the enactive notion of cognition in general; and *participatory sense-making*—enactive social cognition. Along the way, important concepts to pick up are *autonomy* (both as applied to individuals and to social interaction processes), *embodiment*, *experience*, *coordination*, and *rhythm capacity*.

### Sense-making

Enactive cognitive science attempts to answer fundamental questions such as: what is an agent, what is autonomy, why do cognizers care about their world, why does anything mean something to someone? Enaction is a non-reductive naturalistic approach that proposes a deep continuity between the processes of living and those of cognition. It is a scientific program that explores several phases along this life-mind continuum, based on the mutually supporting concepts of *autonomy*, *sense-making*, *embodiment*, *emergence*, *experience*, and *participatory sense-making* (Varela et al., 1991; Thompson, 2005, 2007; De Jaegher and Di Paolo, 2007; Di Paolo et al., 2010).

The organizational properties of living organisms make them paradigmatic cases of cognizers (Varela, 1997; Thompson, 2007). One of these properties is the constitutive and interactive *autonomy* of living systems. This autonomy lies in the fact that they self-generate, self-organize, and self-distinguish. That is, living systems are networks of dynamical processes (metabolic, immune, neural, sensorimotor, etc.) that generate their own identity by self-sustaining and distinguishing themselves from their environment, while at the same time constantly exchanging matter and energy with the environment. An autonomous system is composed of several processes that actively generate and sustain an identity under precarious conditions. In short, living systems are constantly producing themselves physically and regulating their interactions with the world to satisfy the needs created by their precarious condition. Constitutive and interactive properties like these have been proposed to emerge at different levels of identity-generation apart from the metabolic level, including sensorimotor and neuro-dynamical forms of autonomy (Varela, 1979, 1997; Moreno and Etxeberria, 2005; De Jaegher and Di Paolo, 2007; Thompson, 2007; Di Paolo et al., 2010).

Enactive researchers propose that autonomy is also what makes living systems cognizers (Varela, 1997; Weber and Varela, 2002; Di Paolo, 2005; Thompson, 2007). This view rejects the traditional idea that cognizers passively respond to environmental stimuli or satisfy internal demands. Instead, the organism is a center of activity in the world, spontaneously generating its own goals as well as responding to the environment (McGann, 2007). Novel identities emerge, and the coupling between the emergent processes and their context constrains and modulates the operation at underlying levels (Thompson and Varela, 2001; Thompson, 2007; Di Paolo et al., 2010). Actions and their consequences constantly shape the underlying processes and modulate autonomy such that intentions, goals, norms, and significance in general change as a result. The significant world of the cognizer is therefore not pre-given but largely *enacted*, shaped as part of its autonomous activity.

Taking seriously a principle of emergence and mutual constraining between various levels (e.g., personal and subpersonal) makes the enactive approach very skeptical about the localization of function at one level in specific components at a lower level (exemplified in the idea of the homunculus that Frith would like to revive). It rejects “boxology” as a valid method to address the “how does it work” question (De Jaegher and Di Paolo, 2007; Di Paolo, 2009).

For the enactive approach, the body is more than just anatomical or physiological structures and sensorimotor strategies. It is a precarious network of various interrelated self-sustaining identities (organic, cognitive, social), each interacting with the world in terms of the consequences for its own viability. This makes cognition inherently embodied (Sheets-Johnstone, 1999b).

The same applies to experience, which is both methodologically and thematically central for enaction. Experience is not—as it is for cognitivism—an epiphenomenon or a puzzle. It is essentially intertwined with being alive and enacting a meaningful world. Therefore, experience also forms part of the enactive method. It is not just data to be explained, but becomes a guiding force in a dialogue between phenomenology and science, resulting in an ongoing pragmatic circulation and mutual illumination between the two (Varela, 1996, 1999; Gallagher, 1997; van Gelder, 1999).

All these ideas together help us to understand the enactive characterization of cognition as sense-making: a cognizer's adaptive regulation of its states and interactions with the world, with respect to the implications for the continuation of its own autonomous identity. In other words, sense-making is concerned acting and interacting, and the concern comes directly from the sense-maker's self-organization under precarious circumstances. Unlike functionalist approaches, enactivism provides an operational definition of cognition. An organism casts a web of significance on its world. It regulates its coupling with the environment because it maintains a self-sustaining identity or identities that initiate that very same regulation. This establishes a non-neutral perspective on the world. This perspective comes with its own normativity, which is the counterpart of the agent being a center of activity in the world (Varela, 1997; Weber and Varela, 2002; Di Paolo, 2005; Di Paolo et al., 2010; Thompson, 2007). Exchanges with the world are inherently significant for the cognizer. Thus, cognition or sense-making is the creation and appreciation of meaning in interaction with the world. Sense-making is a relational and affect-laden process grounded in biological organization (Jonas, 1966; Varela, 1991, 1997; Weber and Varela, 2002; Di Paolo, 2005; Thompson, 2007). This is why and how things matter to embodied cognizers.

### Participatory sense-making

“Social cognition” understood in enactive terms is better captured by the notion of “intersubjectivity,” which is the meaningful engagement between subjects (Reddy, 2008). Three aspects here are crucial: engagement, meaning, and subject. In the section above, I explained what enactive subjects are, in their inherently meaningful, cognitive-affective interactions with the world. Here, we focus on the encounters between such sense-making subjects.

In order to explain *participatory sense-making* for understanding autism, we need the concepts of (the autonomy of) the social interaction process, engagement, coordination dynamics, and social skills (De Jaegher and Di Paolo, 2007; Fuchs and De Jaegher, 2009; McGann and De Jaegher, 2009; Di Paolo and De Jaegher, 2012), all of which are operational, as I will explain now.

Social interactions are complex phenomena involving verbal and nonverbal behavior, varying contexts, numbers of participants and technological mediation. Interactions impose timing demands, involve reciprocal and joint activity, exhibit a mixture of discrete and continuous events at different timescales, and are often robust against external disruptions. Essential to interaction is that it involves engagement between agents. Engagement (Reddy and Morris, 2004; Reddy, 2008) captures the qualitative aspect of social interactions once they start to “take over” and acquire a momentum of their own. Experientially, engagement is the fluctuating feelings of connectedness with an other, including that of being in the flow of an interaction, and tensions.

We define social interaction on the basis of the autonomy of the interaction process and that of the individuals involved. Thus, a social interaction process is “a co-regulated coupling between at least two autonomous agents, where: (1) the co-regulation and the coupling mutually affect each other, constituting an autonomous self-sustaining organization in the domain of relational dynamics and (2) the autonomy of the agents involved is not destroyed (although its scope can be augmented or reduced)” (De Jaegher et al., 2010, pp. 442–443; also De Jaegher and Di Paolo, 2007, p. 493).

Each agent involved in such a coupling contributes to its co-regulation, but the interaction process also self-organizes and self-maintains. This means that it sometimes continues in a way that none of its participants intends. To illustrate this, think of encountering someone in a narrow corridor. Sometimes, as you meet, in order to avoid bumping into each other, you both step in front of each other a few times, each moving to the same side at the same time—when all you both wanted was to continue on your way. This is a very simple example of the interaction process becoming, for a brief while, autonomous. We defined autonomy above as a self-distinguishing network of processes that sustain themselves under precarious conditions (Varela, 1997; Di Paolo, 2005, 2009; Thompson, 2007). The individual participants as interactors are also autonomous (point 2). If one of them loses their autonomy, for the other it would be like interacting with an object or a tool (De Jaegher and Di Paolo, 2007).

This has a resonance for those with experience of autism. Sometimes a person with autism will take another person by the hand and direct her to something that is out of reach for him. This can feel strange and alienating. The feeling makes sense because, following the definition, this situation would not count as a social interaction, and there would only be a shallow kind of engagement or none at all. One person in the interaction determines the situation (or at least attempts to do so). To neurotypicals, this can be both uneasy and uncanny, because they generally expect even rather instrumental interactions to have some element of mutuality. When this is absent, it is experienced as somehow wrong.

While they last, interactions self-organize and self-maintain through processes of coordination, including its breakdowns and repairs (De Jaegher and Di Paolo, 2007; Di Paolo and De Jaegher, 2012). Coordination is “the non-accidental correlation between the behaviors of two or more systems that are in sustained coupling, or have been coupled in the past, or have been coupled to another, common, system” (De Jaegher and Di Paolo, 2007, p. 490). Coordination is typically easily achieved by simple mechanical means and, when cognitive systems are involved, it does not necessarily require cognitively complicated skill. Coordination can happen at multiple timescales (Winfree, 2001). Temporal coordination is not the only kind; appropriately patterned behaviors, such as mirroring, anticipation, imitation, etcetera are all forms of coordination according to the definition given here. Coordination does not have to be absolute or permanent. There are degrees of coordination and coupled systems may undergo changes in the level of coordination over time (Tronick and Cohn, 1989; Kelso, 1995; Oullier et al., 2008).

Analyses of social interactions and conversations show that participants unconsciously coordinate their movements and utterances (Condon, 1979; Scollon, 1981; Davis, 1982; Kendon, 1990; Grammer et al., 1998; Issartel et al., 2007; Richardson et al., 2007). For instance, listeners coordinate their movements, however small, with the changes in speed, direction and intonation of the movements and utterances of the speaker (Bavelas et al., 2002). Studies of the way musicians play together also show this (see for instance Maduell and Wing, 2007; Moran, 2007). These findings suggest that interactors' perception-action loops are coupled and interlaced with each other (Marsh et al., 2006; Fuchs and De Jaegher, 2009). This includes processes of synchronization and resonance, in-phase or phase-delayed behavior, rhythmic co-variation of gestures, facial or vocal expression, etc. This complexity of interpersonal coordination is already present in early development (Condon and Sander, 1974; Tronick and Cohn, 1989; Malloch, 1999; Jaffe et al., 2001; Stern, 2002/1977; Trevarthen and Malloch, 2002; Malloch and Trevarthen, 2009).

We coordinate in different modalities (movement of different parts of our bodies, gestures, language, thoughts, etc.). We can distinguish a range of different kinds of coordination, such as pre-coordination, one-sided and bi-directional coordination (Fuchs and De Jaegher, 2009). Patterns of interpersonal coordination can directly influence the continuing disposition of the individuals involved to sustain or modify their encounter (De Jaegher and Di Paolo, 2007; Oullier et al., 2008). This is due to the fact that the interactors, generally, are highly plastic, and susceptible to being affected by the history of coordination. When this double influence is in place (from the coordination onto the unfolding of the encounter and from the dynamics of the encounter onto the likelihood to coordinate), we are dealing with a social interaction. This emerging level is sustained and identifiable as long as the processes described (or some external factor) do not terminate it.

With the concept of coordination and other dynamical systems tools, interaction dynamics can be measured (see e.g. Kelso, 2009a,b). Moreover, they can be related to neural activity. The field of second-person neuroscience is growing (Schilbach et al., in press) and the investigation of people interacting live has produced interesting results (e.g. Lindenberger et al., 2009; Dumas et al., 2010, 2012; Cui et al., 2012; Konvalinka and Roepstorff, 2012). This is a welcome development and we have formulated enactive proposals of what taking the interaction process seriously means for understanding brain mechanisms involved in social interactions (Di Paolo and De Jaegher, 2012).

The consequence of these developments for social understanding—and here we come to the concept of *participatory sense-making—*is that, when we engage in interaction, not only the participants, *but also* the interaction process as such modulates the sense-making that takes place. This means that intentions can be truly understood as generated and transformed interactionally. Sometimes, it is impossible to say who is the “author” of the intention, whether it be an emotion, a thought, a belief, or something else. Interacting with each other thus opens up new domains of sense-making that we would not have on our own. There are, moreover, degrees of participation; we sometimes participate a lot (joint meaning-making) and sometimes minimally (one-sided coordination, where, for instance, we point out an object or an idea to someone).

With this view comes a particular approach to social skill (McGann and De Jaegher, 2009). Social skill is evidenced in interactive performance that cannot be conceived purely as an individual feat. Social skill is the flexibility to deal with the regularities (and irregularities) of the social domain provided by the actions of others. This flexibility, though partly determined individually, is also determined by the process of interaction. Moreover, social skills involve “acting through socially constructed norms and practices” (ibid. p. 430). These societal norms and practices are coordinated and negotiated in interaction with others, “rather than simply acted out without sensitivity to the actions of the other” (ibid. p. 431).

Specifically, as regards timing and coordination, one aspect of social skill is what we call the *rhythm capacity* (De Jaegher, 2006). This is the skill to flexibly switch between different interaction rhythms, or “a mastery of mutual coordination” (McGann and De Jaegher, 2009, p. 431). The notion of social skill can be applied to an individual interactor by considering his performance along a certain scale of interest across different interactions, and can be tested, for example, by investigating range of flexibility.

The rhythm capacity is only manifested in interaction processes. It is always also dependent on other interactors' behaviors and the dynamics of the interaction processes. In contrast to an individual skill like typewriting, the rhythmic capacity is also dependent on a relation of mutuality and coherence with the social skill of other interactors involved. It is impossible to test this in the absence of another person who also brings their own rhythmic capacity, and the interaction between them. The performance of rhythmic capacity is partly determined by the interaction process. It can be empirically measured in terms of frequencies and timescales of recoveries from coordination breakdowns (e.g., infrequent breakdowns and/or fast recoveries would be indicative of a high rhythm capacity).

In short, the argument for participatory sense-making is this: If, as indicated above, we make sense of the world by moving around in it and with it (sense-making is thoroughly embodied), and we coordinate our movements with others when interacting with them, this means that we can coordinate our sense-making activities, affecting not only how we make sense of the world but also of others and of ourselves. That is, we literally *participate in each other's sense-making*. We generate and transform meaning together, in and through interacting.

## Sense-making and participatory sense-making in autism

The enactive approach to autism considers the particular difficulties of sense-making and participatory sense-making in autism. Underlying sense-making in general are a person's organismic self-organization, embodiment, needs, skills, and situation. In section “Evidence for a Different Sense-Making in Autism,” we delve into aspects of sense-making in autism, on the basis of evidence from studies of autistic perception, movement, and affect. Differences in these domains, I propose, correspond with a different enactment and understanding of the world. In section “Participatory Sense-Making in Autism,” we discuss how this works in the social realm, where a further important factor is the interaction process, and take a look at participatory sense-making in autism. In each area, I propose novel hypotheses for further research.

### Evidence for a different sense-making in autism

Here, I review evidence for what I call autistic embodiment, i.e., the particular ways in which the biology, neurophysiology, affective, and sensorimotor structures and skills of people with autism differ from those of non-autistics.

Current research investigates “autistic embodiment” as if it consisted of distinct parts. Perception is mostly studied separately from movement and affect, and different pre-supposed sub-aspects of each (e.g., feature detection, categorization, pattern recognition; movement planning and execution; expression and recognition of emotion) are investigated in isolation from each other (see e.g., Rinehart et al., 2001, 2006; Gowen and Hamilton, 2013). Questions that dominate research on sensorimotor aspects of autism are: which kind of processing is primary (e.g. “low-level” vs. “high-level”); what are the differences between autistic and non-autistic perception, movement, and sensation; are we dealing with underperformance or with superior performance; is the connection between motoric/perception particularities and the social/emotional aspects of autism one of correlation, precedence, causation, or amplification (e.g. Happé, 1999; Mottron et al., 2006; Papadopoulos et al., 2012). There is no agreement on whether people with autism are indeed “differently embodied” and if so, precisely how, but research on these matters is on the rise (Leary and Donnellan, 2012; Donnellan et al., 2013).

Often, the particularities of the ways in which people with autism behave are seen as disturbed or disruptive and consequently as “to be treated away.” Two questions *not* generally asked in current research are: *whydo people with autism move and perceive in the way that they do*, and *what does this have to do with how they engage with and understand the world, others, and themselves*? If we consider embodiment and sense-making as fundamentally interwoven, these questions are basic. When a person with autism moves, perceives, or emotes differently, this relates inextricably to how he understands the world. This fact is under-recognized in research that considers perceptual, motor, and affective behaviors in view of their role in the functional whole of cognition, instead of in relation to what matters to the person. We need to find out the precise link between sensorimotor-affective characteristics of autism and the way in which autistic people make sense of their world (Savarese, 2010; Robledo et al., 2012; Torres, 2012; Donnellan et al., 2013).

I propose that the notion of sense-making—integrative as it is of perceptual meaning and affective value—is particularly well-placed to interpret the wide-ranging evidence on the sensorimotor-affective aspects of autism. The concept of sense-making may also help integrate the evidence into a comprehensive, coherent framework that can generate further refined research hypotheses^4^.

#### Perception, movement, and affect in autism

Autistic perception was a legitimate area of study in the 1960s (see e.g., Rimland, 1964; Hermelin and O'Connor, 1965, 1970; Frith and Hermelin, 1969). In 1987, Frith and Baron-Cohen asserted that there were no low-level perceptual problems in autism, and that perceptual differences were due to cognitive processing deficits (Frith and Baron-Cohen, 1987). This is also a basic assumption of the WCC theory, which Frith proposed a few years later in recognition of those aspects of autism that could not be easily explained by ToM, like the islets of ability or the attention to detail (Frith, 1989). While WCC inspired a shift in research focus toward autistic perception, including investigations of so-called low-level perception (Happé, 1996), it considers perception as regulated, top-down, by cognitive processes and thus these cognitive processes as central (Happé and Frith, 2006). Therefore, even if WCC put autistic perception on the research map, its focus is on cognitive processing.

While sensory and perceptual differences are not considered centrally in the main explanatory theories of autism introduced above, they feature prominently in many autobiographical accounts (Williams, 1992; Grandin, 1995; Sacks, 1995; Gerland, 1996; Chamak et al., 2008; Robledo et al., 2012). Everyday sensations that non-autistics generally are not aware of, like the touch of the fabric of a pair of new trousers on the skin, can hurt people with autism. Some loud noises, especially sudden ones, may be unpleasant, while others are pleasurable. Autistic people may not notice other people talking to or touching them, thus being hypo-sensitive to particular events. There are no general patterns of hyper- or hypo-sensitivity, and sensory responses vary greatly across the spectrum, and manifest both toward social and non-social stimuli (Baranek, 2002; Rogers and Ozonoff, 2005; Kern et al., 2006).

Sensory sensitivity has been linked to problems with attention and attention-shifting (Liss et al., 2006). Attention-shifting has been found to be slower in autism than in the non-autistic population (Casey et al., 1993; Courchesne et al., 1994; Townsend et al., 1996), and Liss et al. (2006) hypothesize that hyper- and hypo-sensitivity are due to a decreased ability to modulate attention (see also Landry and Bryson, 2004). It would therefore seem to be a kind of strategy to deal with overstimulation (Markram et al., 2007; Markram and Markram, 2010).

Research suggests that children with autism perceive visual motion differently. Gepner et al. (1995), for instance, found that children with autism have a weaker postural response to the perception of movement compared to non-autistic children, especially when the movement is very fast (Gepner and Mestre, 2002a). Gepner and Mestre (2002b) also propose that there is a “rapid visual motion integration deficit” in autism, manifesting, for instance, in rapid blinking or looking at things while moving the fingers rapidly in front of the eyes (see also Williams, 1992). Gepner and Mestre propose that the “world moves too fast” for children with autism, and that this is why they need to “slow it down” by exploring it in ways like those just mentioned. One of their experiments suggests that the effect of the rapid, rhythmic, involuntary eye-movements when perceiving fast-moving objects (optokinetic nystagmus, this happens for instance when looking outside while you are in a fast train) is weaker in autistic than in non-autistic children (Gepner and Massion, 2002; Gepner and Mestre, 2002b). Furthermore, people with autism find it easier to perceive emotion in moving displays of faces when the images are shown slowed down^5^ (Gepner et al., 2001). Research suggests that autistic people have a higher threshold for perceiving motion coherence (Milne et al., 2002), direction of motion (Bertone et al., 2003), and biological motion (Blake et al., 2003 and Klin et al., 2009). Gepner and Mestre (2002b) also propose possible underlying neurological mechanisms, mainly involving the cerebellum^6^. The research by Gepner and colleagues combines insights into autistic movement (e.g., postural reactions) with the perception of movement, and thus integrates some aspects of autistic embodiment that fit together also on an enactive logic.

Mari et al. (2003) suggest that movement problems should be considered basic to autism. They investigated “reach-to-grasp movement” and found that children with autism had more difficulties in planning and execution than the non-autistic control group. Leary and Hill (1996), in their review article on movement disturbances in autism, also argue that movement difficulties should be seen as core to the condition and that they are at the basis of the social difficulties of people affected. According to them, movement difficulties in autism include problems of movement function such as posture, muscle tone, non-goal directed movements such as nervous tics and action-accompanying movements, and difficulties with voluntary movements, which implicate language and movement planning. Papadopoulos et al. (2011, 2012) and Bhat et al. (2011) provide recent supporting evidence.

There is no real agreement on the extent and kinds of sensorimotor disturbances in autism. Several kinds of impairments have been found, and a variety of causes indicated (Vilensky et al., 1981; Jones and Prior, 1985; Bauman, 1992; Hallet et al., 1993; Gepner et al., 1995; Haas et al., 1996; Rapin, 1997; Ghaziuddin and Butler, 1998; Teitelbaum et al., 1998, 2004; Turner, 1999; Brasic, 2000; Müller et al., 2001; Rinehart et al., 2001, 2006; Gepner and Mestre, 2002b; Schmitz et al., 2003; Martineau et al., 2004; Bhat et al., 2011; Dowd et al., 2012). In contrast to this, Minshew and her colleagues did not find low-level sensorimotor deficits in autism (Minshew et al., 1997, 1999). Fournier et al. (2010) recently reviewed the literature on motor coordination deficits, and conclude that they are “a cardinal feature of ASD” (p. 1227). Other research suggests that people with autism have difficulty combining tasks that require perceiving and moving in different modalities at the same time (Bonneh et al., 2008; Hill et al., 2012). Mottron et al. (2006; Mottron and Burack, 2001) propose that there is an enhanced perceptual functioning in autism.

#### From embodiment to sense-making

Since embodiment and sense-making are intrinsically connected, the body partly determines how we interact with the world. “The world” is moreover that of a specific agent—not that of an external observer. That is, in the way you relate to the world, you construct and pick up as relevant that which is meaningful to you, but not necessarily to someone else. Sensory hyper- and hypo-sensitivities and particular patterns of moving, emoting, and perceiving influence autistic sense-making, and vice versa. In general, the sensorimotor and affective aspects of autism can be seen as alternative ways of perceiving the world or also as strategies to cope with it, for instance in order to slow down the world, or to avoid or modulate stimuli that switch quickly in rhythm and pattern.

Sense-making is a narrowing down of the complexity of the world. Non-autistic sense-making often ignores certain details and jumps to a particular significance (I'm thirsty, I want water, I get it but hardly care about whether the glass is tall or short, transparent, opaque, etc.). People with autism often perceive more detail, but to the detriment of not perceiving quickly enough that which is more salient in a non-autistic context (for instance, when a person with autism grabs someone else's glass of water and drinks from it, not noticing whether this is appropriate or not in the social context, Vermeulen, 2001).

If autistic embodiment is intrinsically linked with autistic sense-making, we can hypothesize that many autistic people will find joy or significance in behaviors and embodied styles of sense-making that are considered “autistic.” An often-ignored factor in perception is the aesthetic element. There may be a value to some autistic sense-making which is simply that of enjoying or remarking on patterns—patterns in space, in ideas, in numbers, in size, in time. Rich patterns exist everywhere in the world, and many autistic people value them, care about them, even enjoy them. This makes ignoring the pattern or the detail doubly difficult. People with autism not only do not initially or without prompt or necessity perceive holistic meaning, but they may feel that they will lose something salient if they (are made to) try to capture the gist of something.

The enactive approach conceives of the way people with autism perceive, make sense, move, and emote, as intrinsically meaningful to them. In this, autistic people are no different from other people. An easy way to test this idea is to see whether persons with autism enjoy or suffer from that which they do and which seems strange to non-autistics.

For instance, in relation to their compulsion for detail, we can ask whether people with autism are, in general, at ease with their disposition for piecemeal processing. Do they regret missing the holistic sense or pity non-autistics for not enjoying detailed patterns? If the hypothesis is true, people with autism can be properly described as having a different conception of wholeness, one that has to do with order, patterns, exceptions, and perceptual richness. Anecdotal evidence for this idea comes from aesthetic appreciation, savant skills, and creativity in autistic people (see e.g., Sacks, 1985; Happé and Frith, 2009). Stronger evidence for WCC having a potential value or significance for people with autism is harder to find. WCC has been described positively as a cognitive style (Happé, 1999), and Happé and Frith (2009, p. 1348) suggest that there is a “rage to learn” and an intrinsic motivation in special talents, indicating that the special skills, as well as their learning and the learning of certain information can be interesting in their own right.

However, savant skills and high creativity are not representative of the whole autistic population (Hacking, 2009). Also, most of this research is concerned with how the processing style relates to other isolated aspects of the functioning of the person with autism, not with their personal significance or more general value. What enaction predicts goes beyond the cognitivist conception in which functioning and adaptation are considered as adequate fit to the non-autistic context. Enaction is concerned with functioning as valued and significant from the perspective of the person herself, in her context. Cognitive, perceptual, sensorimotor, and affective styles should in the first instance be approached from the point of view of the situated self-organizing sense-maker, not just that of an “objective” observer. What is such an observer objective about if he studies cognition but misses the *meaning for the subject* whose cognition he is studying?

An area in which there is evidence that people with autism derive pleasure from their specialized activities or thinking styles is restricted interests and repetitive behaviors. Circumscribed interests are highly frequent in autism, with between 75–88% of the autistic population engaging in them (Klin et al., 2007; Spiker et al., 2012). In direct support of the enactive hypothesis, repetitive activities in autism—unlike obsessions and compulsions in obsessive compulsive disorder—have been found to be “beloved activities apparently associated with great positive valence” (Klin et al., 2007, p. 97; see also Baron-Cohen, 1989; Klin et al., 1997). It has been found that circumscribed interests are highly motivating for children with autism, and that allowing them to engage in these behaviors can help them produce appropriate behaviors (Hung, 1978), and increase social interactions with non-autistic peers and with siblings (Baker et al., 1998; Baker, 2000). Lovaas also considers repetitive interests as intrinsically motivating for the perceptual reinforcement and self-stimulation that they provide, even connecting this to the sensory joys of gourmet food, art, recreational drugs, and smoking (Lovaas et al., 1987).

In a qualitative interview assessing how people with autism and their siblings and parents experience the restricted interests, Mercier et al. (2000) found that they “provide a sense of well-being, a positive way of occupying one's time, a source of personal validation, and an incentive for personal growth” (p. 406). The interviewees also recognized negative aspects of repetitive and circumscribed activities, such as their invasiveness, the amount of time they occupy, and (fear of) potentially socially unacceptable behaviors they may provoke. One of the participants sums up the tension between the positive and negative aspects as follows: “Basically, what others will tell me is that I monopolize time that could have been used for better things. But sometimes I can't think of better things to do when I have my free time” (p. 414).

In contrast to Mercier et al.'s subject-oriented approach, a recent study attempted to show a link between anxiety and restricted interests based on the assumption that restricted interests are a (maladaptive) way of coping with distress (Spiker et al., 2012). The study found that particular kinds of restricted interests were associated with anxiety, while others were not. However, the kind that was associated with anxiety, viz. “symbolically enacted restricted interests,” is not defined or even described in the paper. Moreover, the authors themselves say that it might be that “symbolically enacted RI [restricted interests] only appear coupled to anxiety in children with high functioning ASD because these problems have overlapping behavioral manifestations, such that RI-related behaviors may be misinterpreted as anxiety-related behaviors” (ibid. p. 316). Furthermore, unlike in Mercier et al.'s (2000) study, the nature and incidence of restricted interests was gathered from interviews with the parents, not with the children themselves, and all the children involved in the study were diagnosed as having an anxiety comorbidity (thus biasing the answer to the question of a relation between anxiety and restricted interests in the cases studied).

Restricted interests, focus on detail, and other autistic sensorimotor and affective particularities often interfere with everyday life, and this can make them difficult to deal with, both for the person with autism and for their social and familial environment. However, this does not imply that they could not in themselves be relevant, salient, or significant for the person with autism. It might be that these behaviors are disruptive as a consequence of their manifesting in a context that can or will not accommodate them. This is not to suggest that such behaviors should simply be accepted. Rather it is to suggest that dealing with them should also start from the meaning they have for the person with autism, not just from the question of whether they are appropriate. The interviews conducted by Mercier and colleagues show that doing this can help find suitable ways to deal with the restricted, repetitive behaviors, even to the point of converting them into acceptable activities or extinguishing them (Mercier et al., 2000).

### Participatory sense-making in autism

Participatory sense-making relies on the capacity to flexibly engage with your social partner from moment to moment, where this engagement involves emotion, knowledge, mood, physiology, background, concepts, language, norms, and, crucially, the dynamics of the interaction process and its coordinations and breakdowns. I have conjectured that a sensorimotor interactional coordination ability is at the basis of this connection.

We have seen that sensorimotor differences imply a different sense-making in autism. Sensorimotor differences, especially those involving temporal aspects of perception and movement, will affect interaction and coordination in social encounters, and therefore introduce systematic differences in participatory sense-making. This is true the other way around as well. If social connection is basic to individual cognitive/emotional development (Hobson, 2002), embodiment and sense-making will be influenced by a history of interactive engagements. In the following, I paint an increasingly inter-individual picture of (social) sense-making in autism and its problems.

#### A differently salient social world

Different aspects of the social environment are relevant to people with autism than to non-autistics. Ami Klin suggests that autistic people experience the world, including and especially the social world, as differently salient (Klin et al., 2003). Using an eye-tracker, they analyzed the way persons with autism scan film scenes in comparison with neurotypicals. Autistic people looked significantly less at socially salient aspects like the eyes and mouths of protagonists, or the object of a pointing gesture than non-autistic controls (Klin et al., 2002). It also seems that children with autism do not spontaneously pay attention to social stimuli that are salient to typically developing children, such as human sounds and faces (Klin et al., 2003; Shic et al., 2011). Furthermore, they seem to prefer to attend to inanimate objects over other humans (Klin et al., 2003; Jones et al., 2008). Not only is the preference different, autistic people also seem less sensitive to biological motion, an aspect of the recognition of the motion of other humans (Blake et al., 2003).

Even though Klin and his colleagues emphasize the anchoring of cognition in embodiment and the developmental process of acquiring social cognition, their work still has an individualistic flavor. They hit the nail on the head when they say that “the (non-autistic) child “enacts the social world,” perceiving it selectively in terms of what is immediately essential for social action,” but when they consider the work for this to be done by “perceptually guided actions” (Klin et al., 2003, p. 349), they fall short of the logical next step. They are rightly convinced that social interaction is the basis of social cognition, and they study social capacities from an embodied perspective. The next thing to put up for investigation is the interaction process.

#### Interpersonal engagement in autism

On the enactive account, crucial for social understanding is the capacity to connect. This capacity is relevant both during actual interactions and during non-interactive social situations where social understanding is more observational (Di Paolo and De Jaegher, 2012). If people on the autism spectrum have difficulty connecting, we need to study the social interaction processes they engage in (or fail to engage in).

Peter Hobson argues that, generally, “a conceptual grasp of the nature of ‘minds’… is acquired through an individual's experience of affectively patterned, intersubjectively co-ordinated relations *with* other people” (Hobson, 1993, pp. 4–5, emphasis in original). In other words, social cognition is based in “interpersonal engagement” (Hobson, 2002). With regard to autism, he makes the conjoined claims that what underlies the deficits of autism is a hampered “intersubjective engagement” with social partners from very early in life, and that these engagements are the foundation of flexible and creative thought. Therefore, a deficit in this area would at once explain the problems with social interaction and communication of individuals with autism and the particularities of their ways of thinking (especially literal and decontextualized thinking, well-known to anyone who regularly interacts with people with autism, see also Vermeulen, 2001).

Hobson probes autistic social interactions as they are experienced, to find out how they differ from neurotypical interactions. In this way, he investigates the qualities of relatedness and connectedness. In several imitation studies, he shows that even though children on the spectrum are able to copy actions, they generally do not copy the *way* an action is performed, for instance, whether it was performed harshly or gently (Hobson and Lee, 1999), or directed at the experimenter himself or the child (self- or other-directedness, Meyer and Hobson, 2005). For Hobson and his colleagues, these findings indicate that children with autism identify with others less than typically developing children do: “the autistic individuals were not so much abnormal in their attempts to imitate *the actions* modeled, but instead were abnormal in their attempts to imitate *the person* who modeled” (Hobson and Lee, 1999, p. 657, emphasis in original). What is missing is an imitation of the “expressive quality of another person's behavior” (ibid.).

Interestingly, Hobson also investigated the other side of this: what it is like to interact with someone with autism, in a study called “Hello and Goodbye” (Hobson and Lee, 1998). As the title says, this study analyzes the greetings and farewells of children with autism, compared with a control group of children with learning difficulties. The children were brought into a room to perform a task at a table with an experimenter (Hobson himself), who sat opposite them. The task was no more than a pretext for creating the opportunities for greetings and farewells. Upon entering the room, the children were introduced to Hobson by his colleague. The videotaped episodes of introduction, greeting and farewell were rated by independent judges naïve to the aim of the study, who counted the amount of smiling, nodding, waving and vocalizing of each participant. The hypothesis was supported: children with autism showed fewer greeting and farewell behaviors than the control group, and also combined them less. This is not so surprising given that this result bears out the diagnostic criteria for autism. However, the judges were also given a more subjective item to rate, namely how much interpersonal engagement there was between the participant and the experimenter. They judged that, in the interactions with the participants with autism, there was much less intersubjective engagement at the different stages of the interaction than in those with the non-autistic group.

In a description of this same study in his book *The Cradle of Thought*, Hobson relates something that is not reported in the paper: that, from the videotapes, one could have the impression that, regarding Hobson's own behavior as the interactor, “there was a deliberateness to my own gestures and actions [and that] I was less outgoing and more hesitant in my efforts to make contact, and my ‘Goodbye’ seemed forced. It was clear that I was doing my best to be relaxed and engaging, but I did a poor job when I did not have an engaging partner.” He adds: “The lesson is: interpersonal engagement is just that—interpersonal” (Hobson, 2002, pp. 50–51). For a similar point, made through a study of sharing humor and laughter in autism, see Reddy et al. (2002).

The central issue here—which remains insufficiently investigated—is the interaction process as such. If there are sensorimotor and coordination differences in autism, and we take the embodied interaction process as defined in section “Participatory Sense-Making” as central to social understanding, then we can suspect that the interaction process will be hampered in autism. Is this the case?

#### Interaction rhythm and rhythmic capacity in autism

People with autism often seem awkward in the way they coordinate with others in interactions. Some studies suggest, however, that children with autism have more mastery of the basics of interactional capacity than previously thought. Dickerson et al. (2007), for instance, argue that persons with autism can temporally appropriately place their interventions in social encounters. They investigated interactions between two autistic children and their tutors during question-and-answer sessions involving answer cards, in which both children tapped the answer cards—a seemingly meaningless action. However, Dickerson and colleagues found that the tapping was placed temporally just after the tutor asked the question and before the child started answering, continuing sometimes into the answer of the child. This suggests, first, that the tapping displayed engagement, an engagement that could also have been shown through eye contact, something known to be difficult for people with autism (American Psychiatric Association, 2000). And second, it suggests that the tapping indicated that the child was about to answer the question, i.e., the tapping was “projecting a relevant forthcoming response on the part of the child” (Dickerson et al., 2007, p. 297). Similar findings were made in relation to gaze (Dickerson et al., 2005). Interesting in this research is that the actions of all interaction partners are being investigated, also that of non-autistic participants. This allows to query the experience (cf. Hobson above), as well as the perceived appropriateness of the behavior. The tutors in the tapping study, for instance, took the behavior as interactionally relevant and appropriate (Dickerson et al., 2007).

Other research suggests that people with autism have timing differences. In a study in which participants were asked to tap in synchrony with an auditory stimulus, Sheridan and McAuley (1997) found that the autistic participants' tapping was more variable than that of the non-autistic group (see also Isenhower et al., 2012, for a similar result in an intra-individual bi-manual drumming task). Trevarthen and Daniel (2005) report on interactional timing and rhythmic difficulties in autism in a study of the interactions between a father and his twin daughters, one of whom was later diagnosed with autism (see also St. Clair et al., 2007). With this twin, the father was unable to engage in rhythmic interaction. This is reminiscent of Hobson's Hello and Goodbye study, which also showed that an interaction partner is less able to engage with a partner who is less rhythmically able. Again, it becomes apparent that social capacity is interactional and not just individual.

Another set of investigations centers around the contingency detection hypothesis (Watson, 1979; Gergely and Watson, 1999; Nadel et al., 1999). Gergely (2001) hypothesized that, in normal development, there is a transition from an expectancy of perfect contingency to one of less than perfect contingency. Before they are 3 months old, Gergely conjectures, infants expect to perceive effects of their actions that immediately follow those actions. These are found mostly in their own actions (what Piaget calls “circular reactions,” 1936). Around 3 months, infants start to search for “high-but-imperfect” contingency, which is found in games with other people and in effects of the infant's actions on the environment. With this shift the infant supposedly starts to engage in interactions with the social world. With regard to autism, Gergely reckons that this shift does not take place, or not fully. As a result, the child with autism would continue to seek perfect contingency throughout life. There is no direct evidence for this theory yet, even though it is an interesting hypothesis. Jacqueline Nadel, who has also worked on contingency detection in children both with and without autism, found that children with autism do not spontaneously detect and expect social contingency, although they can learn to do it after an experimental phase in which the adult experimenter has imitated them (see Nadel et al., 2000; Field et al., 2001).

While there is a general and rather vague idea that people with autism are “awkward” in their interactions, until we investigate those interactions, we do not know what this means or entails. If interactional timing is awkward, and one or both partners do not have the flexibility to adapt to the other's timing, the *rhythmic capacities* (see above) will be of a low quality, and this will result in interactional problems. Although further research is needed, the evidence points to various problems with interaction timing in autism, but also unexpected capabilities. On an enactive perspective, both of these will impact on the dynamics of social interaction, specifically on the quality of coordination, the frequency of coordination breakdowns, the ability to repair them, and the experience of the interactors with and without autism, supporting Hobson's observations. Interactions involving people with autism do not fully lack flexibility, but its scope is reduced due to motor and timing differences. This can be both the cause and the symptom of difficulties with connecting. Findings like the ones reported allow to keep searching for and refine hypotheses about what precisely characterizes “autistic interactions.”

One way in which to test rhythmic capacity and other interactional capacities of and with people with autism, is to study how often breakdowns occur, as well as how easily they are recovered from. Dynamical measures of coordination can be used to construct an index of how quickly the pair achieves coordination again after breakdown (see e.g., Kelso, 1995, 2009a,b; van Orden et al., 2003, 2005; Riley et al., 2011). Immediate or fast recovery would indicate a high rhythmic capacity, and slow, absent, “jumpy,” or unclear recoveries would indicate a lower or narrower rhythmic capacity, i.e., little interactional flexibility overall. The prediction is that interactions of people with autism show a marked reduction in rhythm capacity compared to those of non-autistics. Recently, Marsh and colleagues tested this in a study of unconscious rocking (in rocking chairs) between children with and without autism and their parents, finding that children with autism had a lower tendency to rock in symmetrical timing with their parents (Marsh et al., 2013; see also Schmidt and O'Brien, 1997). A similar difference is expected between interactions of people with autism who do or do not have an interaction history with each other (i.e., whether they have interacted before, and how much). The case of interactions between people with autism who have an interaction history is especially interesting, because it brings several predictions together. We predict both that people who have interacted before will have a smoother rhythm capacity, and that people with autism will have a more reduced rhythm capacity. If these two elements come together, i.e., in an interaction between two autistic people with a long interaction history between them, this will have its own specific rhythmic characteristics.

So far, we have discussed interactional capacities, but what about participatory sense-making?

#### What is participatory sense-making like in autism?

Penny Stribling and her colleagues have studied the behavior and speech of autistic children in an interactional context, using conversation analysis. One of their studies evaluates instances of echolalia, produced by a boy with autism in a single session of play with a robot (Stribling et al., 2005/2006). Echolalia is the repetition of utterances (one's own or an other's), and is often considered meaningless and uncommunicative, and the general advice is to ignore it. However, Stribling demonstrates that the repeated utterances of the boy had an interactional function. He repeated a phrase that seemed communicationally irrelevant because of its literal content, yelling ‘spelling assertions’ such as “please has got an A in it!” By taking a panoramic view of the situation, i.e., by studying the utterance in its interactive context, as well as its prosodic characteristics, Stribling et al. found that the boy's supposedly irrelevant utterances were in fact a protest at losing control over the robot, and an attempt to regain it. They suggest this because, first, all the instances of the echoed utterance that they recorded happened when another person was starting to play with the robot, and second, the way the utterances were made had strong prosodic similarities to how a protest generally sounds (rising loudness and emphasis). Further to their explanation, we can add that the utterance could also have an intrinsic meaning. From the enactive point of view, in which a cognizer self-maintains and self-organizes, it can be proposed that the boy is self-affirming his place in an interaction in which he feels that something is taken away from him, by uttering knowledge that he has. These utterances could be a way of maintaining individual autonomy in an interactional situation. This possibility can be further researched using the notions of self-organization and individual and interactional autonomy as conceptual tools for deepening the understanding of phenomena like echolalia.

Difficulties with coordinating and interacting in autism will lead to hampered participatory sense-making because, as we have seen, participatory sense-making is the inter-individual coordination of embodied and situated sense-making. As regards the new domains of sense-making that are generated in interaction it is clear to see that, if there are such difficulties in autistic interaction as I have just described, the range of orientations, from one-sided (or instructive) coordination of a person in their individual cognitive domain to closely coupled mutual orientation of sense-making, will be difficult to achieve. Additionally, because of the experience of negative affect that results from more frequent coordination breakdowns, social interaction may be less often sought by people with autism, resulting in fewer opportunities to engage in participatory sense-making.

One of Hobson's proposals is that flexible thinking develops from affective interpersonal engagement, and that, in autism, hampered interpersonal relating throughout development leads to the cognitive problems of autism, which are characterized by inflexibility of thinking, lack of creativity, and literal and decontextualized understanding (Vermeulen, 2001; Hobson, 2002). Similarly, if, as proposed by Reddy (2003), complex self-conscious emotions develop out of infants' early interactive experiences (in particular the awareness of being the object of another's attention), then a history of non-fluid interactions must impact on the development and understanding of social emotions, such as embarrassment, pride, and shame.

On the present proposal, if the developmental trajectory of participatory sense-making is hindered in specific ways, among others in the area of interactional coordination, this will reinforce a lack of flexibility in thinking and in dealing with self-conscious emotions. In order to specify in detail why this is the case, the present work needs to be extended with a developmental strand. For now, we can conclude that, if there is less flexibility in social interactional timing and coordination, the creation of new domains of sense-making that rely on participation by others is impeded. It is likely that flexibility in both of these areas is strongly related, especially if there is such a strong developmental interaction between them. Further research is needed to find out the precise relationship between interactional flexibility and flexibility in thinking and emoting.

#### Some implications for intervention and diagnosis

Underlying the interactional difficulties of people with autism we could find neurological and/or sensorimotor differences, but such individual differences do not suffice to explain where specific autistic ways of making sense of the world come from. Social understanding is a constitutive aspect of cognition in general, and it is at its basis truly inter-individual (even the personal skills that permit remote observational social understanding, I propose, are dependent on interactive skills and experiences, see Di Paolo and De Jaegher, 2012). Therefore, interventions for autism—w.r.t. social difficulties, cognition, affect, and sensorimotor capacities—need to pay special attention to interactional coordination, rhythmic capacity and participatory sense-making (this is the basis of, for instance, music therapy, and dance and body movement interventions, Wigram and Gold, 2006; Samaritter and Payne, 2013). This is the context that affords the best interpretation of neurological and other individual factors.

Putting things in the appropriate rhythmic and interactive context is not a novelty for many parents, caregivers, teachers, and friends who successfully motivate, adapt to, and engage autistic partners. Such is the case with approaches like Relationship Development Intervention (Gutstein and Sheely, 2002), or intensive interaction (Caldwell, 2006) and similar ones. The gist of these approaches is to gently introduce the child to flexible interactions with both the social and the “non-social” world in playful settings. At the heart of Relationship Development Intervention sits the idea that people with autism have problems with dynamic, but not with static intelligence. The suggestion has been made before that people with autism are good at scientific-style cognition, but have less adaptive, engaged, know-how intelligence (Kanner, 1973; Baron-Cohen, 2002, 2003). The development of flexibility in interaction can aid the development of flexibility and creativity in behavior and thinking in general, as the present work also predicts, in line with Hobson's ideas (Hobson, 2002), and enhance daily support, friendships, and love relationships.

## Conclusion

In this paper, I have looked at autism through an enactive lens in order to help integrate the diverse aspects of autism that have up to now been examined in isolation. Unlike the search for a common root or key causal factors, enaction strives for a coherent picture of autism, while embracing a complex, non-linear multi-causality. In this effort, two elements that I aimed to do justice to are the experience of autism—both that of people with autism and that of those interacting with them—and the differences in embodiment that seem present in autism.

I suggest that people with autism make sense of the world differently, and that, in the social realm, they are differently able to participate in sense-making with others.

This leads to the following methodological considerations. If we base autism research on the question of why something means something for someone, we can connect autistic styles of sense-making with particular ways of moving, perceiving, and emoting. Hypotheses based in a subject-oriented approach to cognition and mind in autism will be better able to connect the elements that up to now have remained disconnected. For instance, I proposed that restricted interests and repetitive behaviors, if given a place in the actions and interactions of people with autism, can help them, among other things, to improve their social flexibility. I suggested that a focused treatment is needed of a surprising blind spot in autism research: the social interaction process itself. Once we do that, we will be better able to understand both the difficulties and the capacities that people with autism have in this domain. Behaviors that seem irrelevant can acquire significance from the context of the social interaction. To understand this, we must abandon disembodied individualism.

I have hinted at the possible developmental questions that may arise from considering both subjective and interactive factors. This is one of the directions where further work is needed. Another such open direction is to draw further implications for diagnosis, therapy, and interventions.

Ethically, the approach put forward here is not one of *laissez faire*. On the contrary, it is one that starts from *also* taking seriously the perspective and subjectivity of people with autism themselves, in a principled, coherent, and comprehensive way. It is then that we can expect to be able to build bridges that are well-informed by both autistic and non-autistic experience.

### Conflict of interest statement

The author declares that the research was conducted in the absence of any commercial or financial relationships that could be construed as a potential conflict of interest.

## Footnotes

^1^“Neurotypicals” is a term used by autistic people to denote non-autistics, see http://isnt.autistics.org/ (a satirical website by people with autism), http://www.autistics.org/ (a site by people with autism for people with autism) and http://en.wikipedia.org/wiki/Neurotypical. I use this term interchangeably with “non-autistics.”

^2^Which in itself is a problem. The research focus on children can affect the actual lives of people with autism throughout life. In many countries, care facilities are directed at children, and there are no services for those over 18. This is the case in the two countries where I have had experience with autism facilities, Belgium and the UK, and it is possibly similar in other countries.

^3^And even physical therapists call for the development of more embodied interventions (Bhat et al., 2011).

^4^Note that my use of the term sense-making differs from that of Noens and van Berckelaer-Onnes (2005). They use the notion in the context of the WCC theory, without defining it. Sense-making, in their usage, is to do with “meaning perception,” for instance in organizing elements of perception into a functional whole (e.g., seeing a gestalt), and sometimes, with communication, as in “the exchange of meanings” (Noens and van Berckelaer-Onnes, 2004, p. 202). Their use of the term has none of the enactive background in terms of embodiment, experience, self-organization, and autonomy, and is not connected with a wider sense of subjectivity, even if, again, their ultimate concern is with providing a better fit between the person with autism and his world.

^5^This, on a cognitivist account, could be said to be because they have an explicit ToM approach to emotions, i.e., because they have to think about and infer what the emotions are. The argument would be that this is a slower process than emotion recognition in neurotypicals, and that this is the reason why it is easier like this for them. An enactive account would conjecture that they do not have the interactive experience, and that this is why, indeed, they may have to “figure out” the emotions, rather than relate to them via connection, interaction processes, “direct perception” (Gallagher, 2008), and participatory sense-making.

^6^The role of the cerebellum is very relevant, and a possibly fruitful topic for future research, as it is implicated in movement and timing.
